# Supplementary material for: A Humanized Yeast Phenomic Model of Deoxycytidine Kinase to Predict Genetic Buffering of Nucleoside Analog Cytotoxicity
Source: Genes (Basel). 2019 Sep 30;10(10):770. doi: 10.3390/genes10100770 (PMC6826991; doi:10.3390/genes10100770)
Supplement: Supplementary file 1 [file genes-10-00770-s001.zip › Additional_Files/Additional_File7_GOTermHeatmaps.zip!/Additional_File7_GOTermHeatmaps/nuclear_pre-replicative_complex.pdf]

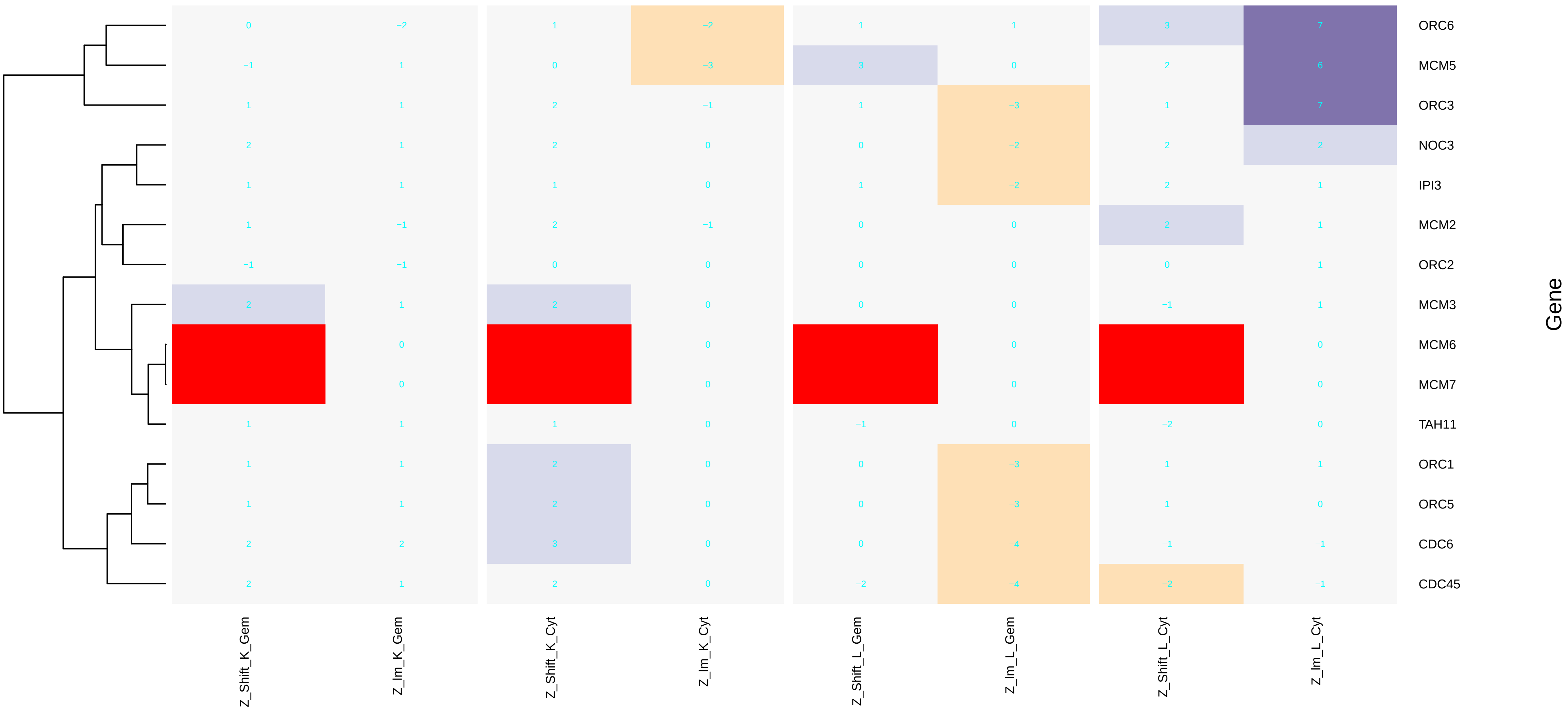

ORC6
MCM5
ORC3
NOC3
IPI3
MCM2
ORC2
MCM3
MCM6
MCM7
TAH11
ORC1
ORC5
CDC6
CDC45
Gene
Z_Shift_K_Gem
Z_Im_K_Gem
Z_Shift_K_Cyt
Z_Im_K_Cyt
Z_Shift_L_Gem
Z_Im_L_Gem
Z_Shift_L_Cyt
Z_Im_L_Cyt
0 −2 1 −2 1 1 3 7
−1 1 0 −3 3 0 2 6
1 1 2 −1 1 −3 1 7
2 1 2 0 0 −2 2 2
1 1 1 0 1 −2 2 1
1 −1 2 −1 0 0 2 1
−1 −1 0 0 0 0 0 1
2 1 2 0 0 0 −1 1
0 0 0 0
0 0 0 0
1 1 1 0 −1 0 −2 0
1 1 2 0 0 −3 1 1
1 1 2 0 0 −3 1 0
2 2 3 0 0 −4 −1 −1
2 1 2 0 −2 −4 −2 −1
